# Sulforaphane Attenuates Ethanol-Induced Teratogenesis and Dysangiogenesis in Zebrafish Embryos

**DOI:** 10.3390/ijms252111529

**Published:** 2024-10-27

**Authors:** Zhijian Wu, Shao-Yu Chen, Liang Zheng

**Affiliations:** 1 Department of Pulmonary and Critical Care Medicine, The Second Xiangya Hospital, Central South University, Changsha 410010, China; 2 Department of Pathology and Laboratory Medicine, The University of Kansas Medical Center, Kansas City, KS 66126, USA; 3 Department of Pharmacology and Toxicology, University of Louisville Health Sciences Center, Louisville, KY 40292, USA; 4 Institute of Reproductive and Developmental Sciences, The University of Kansas Medical Center, Kansas City, KS 66126, USA

**Keywords:** sulforaphane, fetal alcohol spectrum disorders, zebrafish embryos

## Abstract

Prenatal ethanol exposure can cause a broad range of abnormalities in newborns known as Fetal Alcohol Spectrum Disorder (FASD). Despite significant progress in understanding the disease mechanisms of FASD, there remains a strong global need for effective therapies. To evaluate the therapeutic potential of sulforaphane (SFN), an active compound extracted from cruciferous vegetables, in preventing FASD, ethanol-exposed zebrafish embryos were pretreated, co-treated, or post-treated with various concentrations of SFN. The FASD-like morphological features, survival rate, hatching rate, and vascular development were then assessed in the zebrafish embryos. It was found that pretreatment with 2 μM SFN during 3–24 hpf had no noticeable protective effects against teratogenicity induced by subsequent 1.5% ethanol exposure during 24–48 hpf. In contrast, co-treatment with 2 μM SFN and 1.5% ethanol during 3–24 hpf significantly alleviated a range of ethanol-induced malformations, including reduced body length, small eyes, reduced brain size, small otic vesicle, small jaw, and pericardial edema. Post-treatment with 3 μM SFN for 4 days following 1.5% ethanol exposure during 3–24 hpf also significantly reduced the characteristic features of FASD, decreasing the mortality rate and restoring body length, eye size, brain size, and otic vesicle circumference. Moreover, we found that ethanol, even at a low dose (0.5%), causes vascular development deficit in the zebrafish embryos, which were also largely rescued by SFN treatment. These data indicated that SFN has great potential to be used in the prevention and treatment of FASD.

## 1. Introduction

Fetal Alcohol Spectrum Disorder (FASD) refers to a range of effects caused by prenatal exposure to alcohol with possible lifelong implications. Fetal Alcohol Syndrome (FAS) is generally considered to be the most recognized form of FASD [1,2]. FAS typically represents facial dysmorphology (i.e., congenital malformation), impaired growth, and cognitive and behavioral abnormalities [3]. The prevalence of the full spectrum of FASD is estimated at 9.1 per 1000 live births in the general population. However, the national rate could potentially be closer to 50 per 1000 in the US and some Western European countries, according to a review of in-school screening and diagnosis studies [4]. The cost of raising a child with an FASD is high. Amendah et al. [5] found that incurred health costs for a child with identified FAS were nine times higher than those for children without an FASD. Moreover, since alcohol-induced teratogenic effects are permanent, an FASD cannot be ‘cured’. To date, success with the typical treatment approach is still lacking.

Sulforaphane (SFN) is an isothiocyanate derived from cruciferous vegetables such as broccoli [6]. It has been considered a promising agent in fighting cancer [7,8,9]. The therapeutic potential of SFN is based on its potent activity in protecting against oxidative stress, inflammation, DNA-damaging electrophiles, and radiation [10,11]. Previous studies have demonstrated that oxidative stress plays an important role in ethanol-induced teratogenesis [12,13] and that ethanol-induced malformations can be partially prevented by using exogenous antioxidants [14,15,16] or by upregulation of Nrf2 (nuclear factor erythroid 2-related factor 2) signaling [17]. Nrf2 is a transcription factor that activates the expression of several antioxidant and detoxifying enzymes, such as glutathione peroxidase and superoxide dismutase [18]. When Nrf2 is upregulated, it helps protect cells from oxidative damage by reducing the accumulation of ethanol-induced reactive oxygen species (ROS) [19]. Notably, SFN has been found to prevent ethanol-induced apoptosis in neural crest cells (NCCs), which are critical for craniofacial development, by activating Nrf2 and its downstream antioxidant proteins [20].

Several animal models, including mouse, rat, chicken, guinea pig, Xenopus, and zebrafish, are utilized to study the structural and functional deficits caused by prenatal ethanol exposure and its underlying mechanisms [12,13,14,16,21,22]. Among them, the zebrafish model has gained widespread acceptance for understanding the pathogenesis of FASD (e.g., [22,23,24]). To date, there are very limited treatment options available for FASD. Current approaches primarily focus on early intervention and supportive therapies to manage symptoms and improve quality of life [25,26]. The aim of this study is to evaluate the effectiveness of SFN in reducing ethanol-induced teratogenesis in zebrafish embryos. Our results demonstrated that treatment with SFN can diminish ethanol-induced growth retardation, malformations, and dysangiogenesis in zebrafish.

## 2. Results

### 2.1. The Temporal Window of Ethanol Sensitivity in Zebrafish Embryos

**Figure 1:**
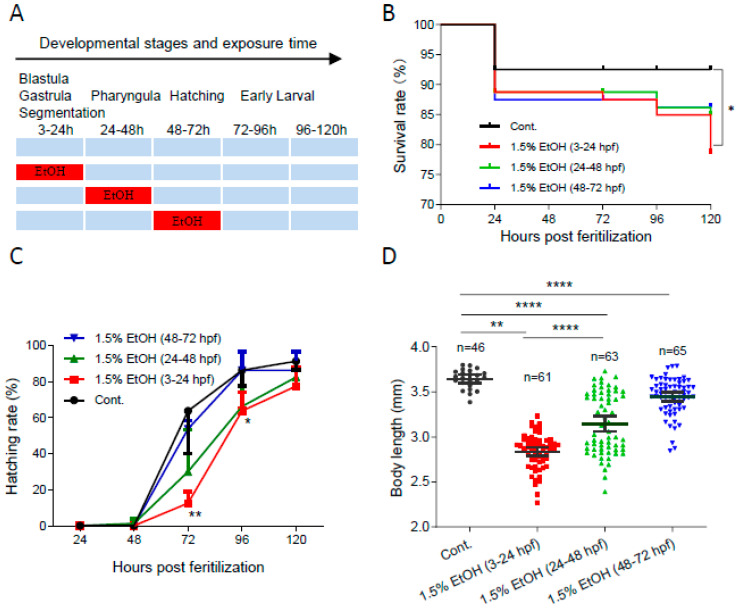
**The temporal window of ethanol sensitivity in zebrafish embryos.** (**A**) Zebrafish embryos were exposed to 1.5% ethanol during either 3–24 hpf, 24–48 hpf, or 48–72 hpf, then rinsed and incubated in egg water. The survival rate, hatching rate, and body length of exposed groups and control were indicated in (**B**–**D**), respectively. One-way ANOVA was used to compare the ethanol exposure groups to the control with * *p* < 0.05, ** *p* < 0.01, and **** *p* < 0.0001.

To determine the temporal window of ethanol sensitivity in zebrafish embryos, the embryos were exposed to 1.5% ethanol at different developmental stages (Figure 1A). As shown in Figure 1B, ethanol exposure increased the mortality rate in zebrafish embryos at various developmental stages. The mortality rate at 120 hpf significantly increased by 2.8-fold in embryos exposed to 1.5% ethanol from 3 to 24 hpf. However, ethanol exposure during other tested periods, 24 to 48 hpf or 48 to 72 hpf, did not result in a significant decrease in survival rate. Additionally, ethanol exposure decreased the hatching rate, with the lowest hatching rate observed in embryos exposed to ethanol from 3 to 24 hpf (Figure 1C). While ethanol exposure reduced the body length of embryos in all treated groups, the shortest body length (2.84 ± 0.19 mm) was observed in zebrafish embryos exposed to ethanol from 3 to 24 hpf (Figure 1D). These results indicate that zebrafish embryos are most sensitive to ethanol-induced embryotoxicity during the 3 to 24 hpf window, which corresponds to the gastrulation and somitogenesis stages.

### 2.2. Pretreatment with SFN Does Not Diminish Ethanol-Induced Embryotoxicity in Zebrafish

**Figure 2:**
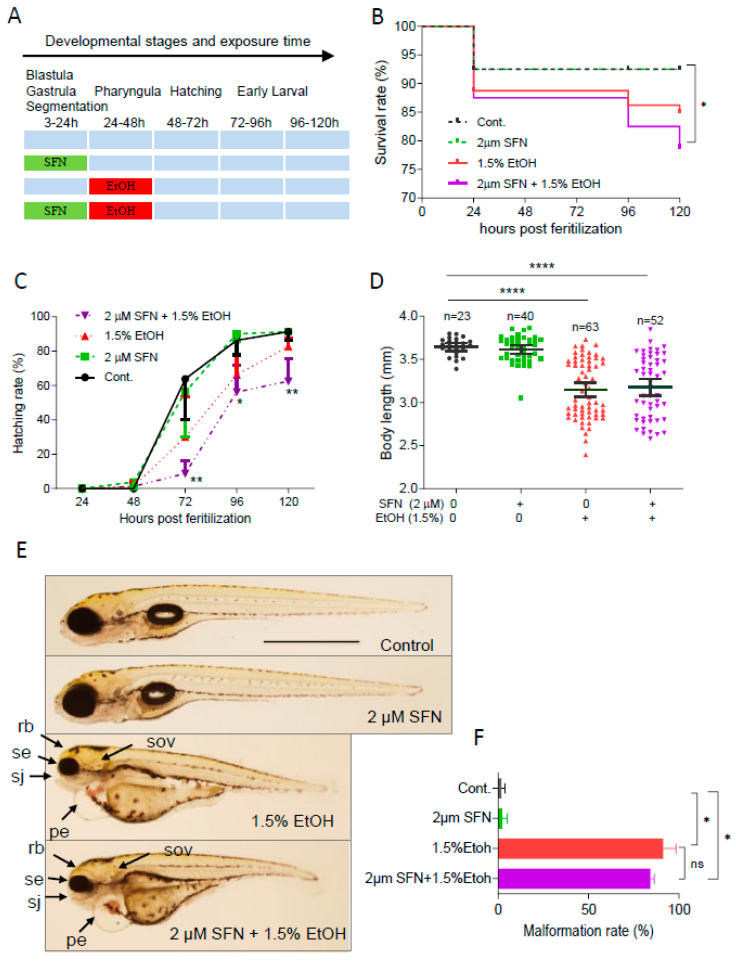
**Pretreatment with SFN showed no protective effect against ethanol-induced teratogenicity.** (**A**) Zebrafish embryos were treated with 2 µM SFN from 3 to 24 hpf, then exposed to 1.5% ethanol from 24 to 48 hpf, and thereafter incubated in fresh egg water. (**B**) Survival rate. (**C**) Hatching rate. (**D**) Body length. (**E**) Representative larvae of control without treatment (first row), 3–24 hpf treatment with 2 µM SFN (second row from top), 24–48 exposure with 1.5% ethanol (third row), and 3–24 hpf pretreatment with 2 µM SFN and then 24–48 exposure with 1.5% ethanol (bottom row). (**F**) Histogram (next to the larvae images) indicates the percentage of larvae with typical FASD malformations. Arrows indicate the phenotypes of FASD. se, small eyes; rb, reduced brain; sov, small otic vesicle; sj, small jaw; and pe, pericardial edema. One-way ANOVA was used to analyze the effects of SFN alone and *t*-test for pairwise comparison with ns, not significant, * *p* < 0.05, ** *p* < 0.01, and **** *p* < 0.0001. The scale bar represents 1 mm.

To determine whether pretreatment with SFN can prevent ethanol-induced toxicity in zebrafish embryos, the embryos were treated with 2 μM SFN from 3 to 24 hpf followed by exposure to 1.5% ethanol for 24 h from 24 to 48 hpf (Figure 2A). The result showed that pretreatment with 2 μM SFN alone did not reduce the ethanol-induced mortality rate in zebrafish embryos (Figure 2B). Pretreatment with SFN also did not diminish ethanol-induced reduction in hatching rate. In contrast, pretreatment with SFN resulted in a significant decrease in the hatching rate of ethanol-exposed embryos at 72 hpf (*p* < 0.01), 96 hpf (*p* < 0.05), and 120 hpf (*p* < 0.01) compared to the control (Figure 2C). Additionally, pretreatment with SFN did not mitigate the ethanol-induced reduction in the body length of embryos (Figure 2D). Moreover, SFN pretreatment did not attenuate ethanol-induced dysmorphology in zebrafish embryos. As shown in Figure 2E, small eyes, reduced brain size, small otic vesicles, small upper jaw, and pericardial edema were observed in ethanol-exposed groups with or without SFN pretreatment. Pretreatment with SFN did not reduce the malformation rate in embryos treated with ethanol (Figure 2F). These results indicate that pretreatment with SFN is not sufficient to prevent ethanol-induced embryotoxicity in zebrafish.

### 2.3. Co-Treatment with SFN Protects Against Ethanol-Induced Embryotoxicity

**Figure 3:**
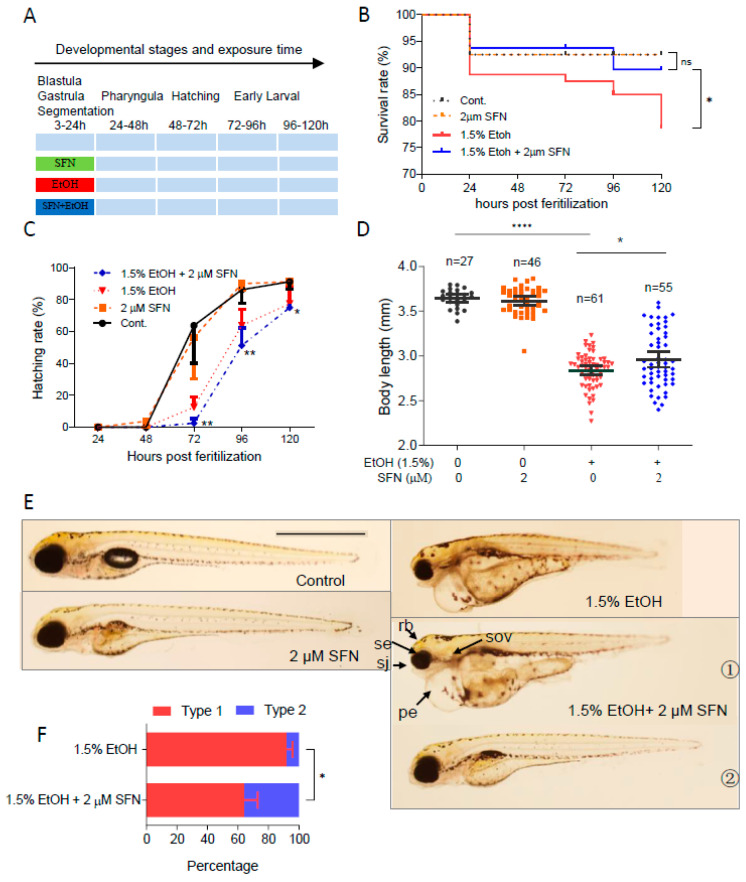
**Co-treatment with SFN protected against ethanol-induced teratogenicity.** (**A**) Zebrafish embryos were co-treated with 2 µM SFN, 1.5% ethanol during 3–24 hpf. (**B**) Survival rate. (**C**) Hatching rate. (**D**) Body length. (**E**) Representative larvae of control without treatment, 3–24 hpf treatment with 2 SFN alone, 3–24 hpf treatment with 1.5% ethanol column, and co-treatment with SFN and ethanol. The larvae in co-treatment groups were classified into two types: type 1 larvae are typically FASD-like, whereas type 2 larvae are more similar to SFN-supplemented control. (**F**) Percentage of type 1 and 2 larvae in the co-treatment groups. Arrows indicate the phenotypes of fetal alcohol spectrum disorder (FASD). se, small eyes; rb, reduced brain; sov, small otic vesicle; sj, small jaw; and pe, pericardial edema. One-way ANOVA was used to analyze the effects of SFN alone and *t*-test for pairwise comparison with ns, not significant, * *p* < 0.05, ** *p* < 0.01, and **** *p* < 0.0001. The scale bar represents 1 mm.

To determine whether co-treatment with SFN can prevent ethanol-induced embryotoxicity, zebrafish embryos were co-treated with 1.5% ethanol with or without SFN from 3 to 24 hpf (Figure 3A). As shown, treatment with 2 μM SFN alone from 3 to 24 hpf had no noticeable effects on the survival rate, body length, or hatching rate of the embryos, indicating the safety of SFN treatment. Strikingly, co-treating ethanol-exposed zebrafish embryos with 2 μM SFN dramatically increased the survival rate compared to that of the ethanol-treated control (Figure 3B). The mortality rate decreased to 10% at 120 hpf in the group co-treated with 2 μM SFN, compared to 21% in the group treated with ethanol alone. Despite this improvement, co-treatment with 2 μM SFN did not prevent the ethanol-induced reduction in hatching rate (Figure 3C). Nevertheless, the body length of the embryos co-treated with 2 μM SFN increased by 4.3% (*p* < 0.05) compared to the group treated with ethanol alone (Figure 3D).

We also found that almost all embryos exposed to 1.5% ethanol from 3 to 24 hpf showed recognizable major characteristics of FASD, including growth retardation, small eyes, and small otic vesicles. Co-treatment with SFN diminished ethanol-induced dysmorphology in zebrafish embryos (Figure 3E). The embryos co-treated with ethanol and SFN could be divided into two types: type 1, where ethanol-induced malformation was not significantly diminished, and type 2, where malformation was significantly attenuated by SFN, as observed in 5 dpf larvae. Type 1 larvae still showed an array of FASD-like dysmorphologies, while type 2 larvae were largely rescued by the co-treatment with SFN, restoring eye size, brain size, pericardial formation with normal fluid homeostasis, and other morphological structures, similar to the control and the group treated with SFN alone. Overall, co-treatment with SFN significantly reduced the malformation rate in embryos compared to the ethanol-treated group. The percentage of type 1 larvae in the group co-treated with 2 μM SFN was significantly less than in the ethanol-treated group (*p* < 0.01) (Figure 3F). These results indicate that co-treatment of SFN protects ethanol-induced teratogenesis in zebrafish embryos.

### 2.4. Post-Treatment with SFN Rescues Ethanol-Induced Embryotoxicity in Zebrafish

**Figure 4:**
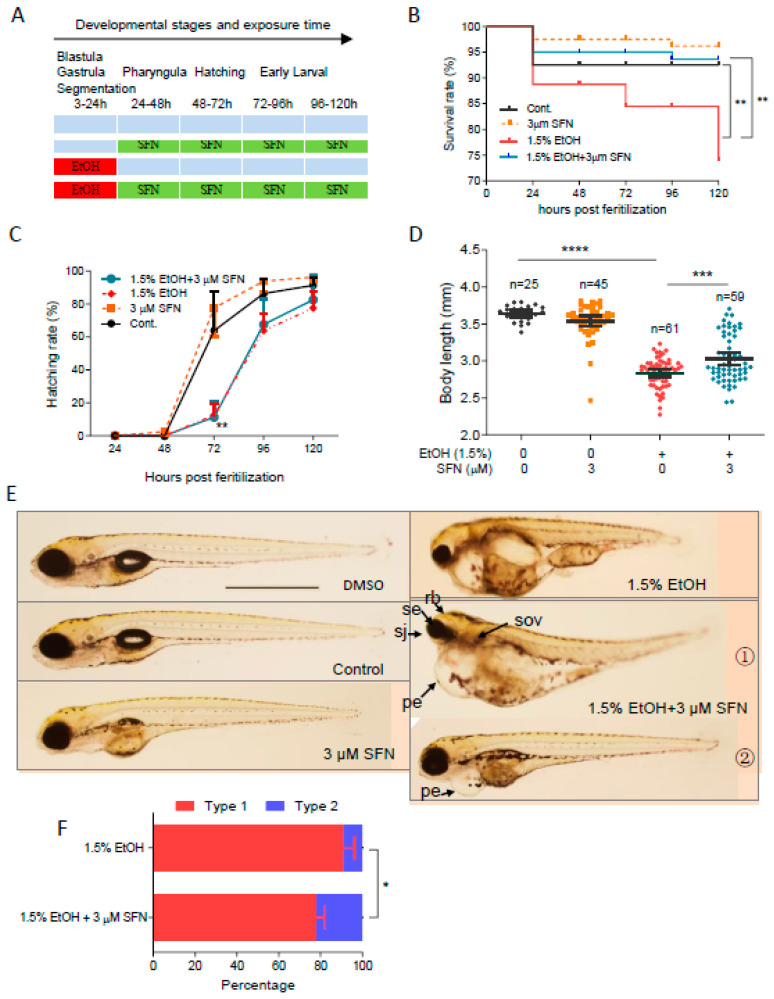
**Post-treatment with SFN also rescued FASD in zebrafish embryos**. (**A**) Zebrafish embryos were first exposed to 1.5% ethanol during 3–24 hpf and then treated with 3 µM SFN for 96 h with renewal every 24 h. Treatment with 3 µM SFN alone with renewal every 24 h and treatment with 1.5% ethanol alone during 3–24 hpf were also performed. (**B**) Survival rate. (**C**) Hatching rate. (**D**) Body length. (**E**) Representative larvae of DMSO control and freshwater control without treatment, 24–120 hpf treatment with 3 µM SFN alone, 3–24 hpf treatment with 1.5% ethanol, and post-treatment with SFN and ethanol. The larvae in post-treatment groups were claasified into two types: type 1 larvae are typically FASD-like, whereas type 2 larvae are more like SFN-supplemented control. (**F**) Percentage of type 1 and 2 larvae in the post-treatment groups. Arrows indicate the phenotypes of fetal alcohol spectrum disorder (FASD). se, small eyes; rb, reduced brain; sov, small otic vesicle; sj, small jaw; and pe, pericardial edema. One-way ANOVA was used to analyze the effects of SFN alone and *t*-test for pairwise comparison with * *p* < 0.05, ** *p* < 0.01, *** *p* < 0.001, and **** *p* < 0.0001. The scale bar represents 1 mm.

To determine whether post-treatment with SFN can attenuate ethanol-induced embryotoxicity in zebrafish, embryos were exposed to 1.5% ethanol from 3 to 24 hpf, followed by treatment with 3 μM SFN from 24 to 120 hpf (Figure 4A). We found that post-treatment with 3 μM SFN reduced the mortality rate at 120 hpf by 70.6% (*p* < 0.05) compared to the group treated with ethanol alone, decreasing from 21.3% to 6.3%. Post-treatment with SFN also significantly diminished ethanol-induced reductions in body length, which increased by 6.8% (*p* < 0.001) in the groups post-treated with 3 μM SFN (Figure 4D). However, post-treatment with 3 μM SFN did not significantly alter the hatching rates in ethanol-exposed zebrafish embryos (Figure 4C). As shown in Figure 4E, post-treatment with 3 μM SFN partially attenuated ethanol-induced eye, brain, otic vesicle, and craniofacial defects. Although edematous pericardium was still notable in these embryos, 22.1% of the total hatched embryos, classified as type 2 larvae, showed significantly larger eye, brain, otic vesicle, and body length compared to the ethanol-exposed larvae. These results indicate that post-treatment with SFN at its optimal dose and interval also effectively reduces ethanol-induced embryotoxicity in zebrafish.

### 2.5. SFN Treatment Attenuates Ethanol-Induced Dysangiogenesis in Zebrafish Embryos

**Figure 5:**
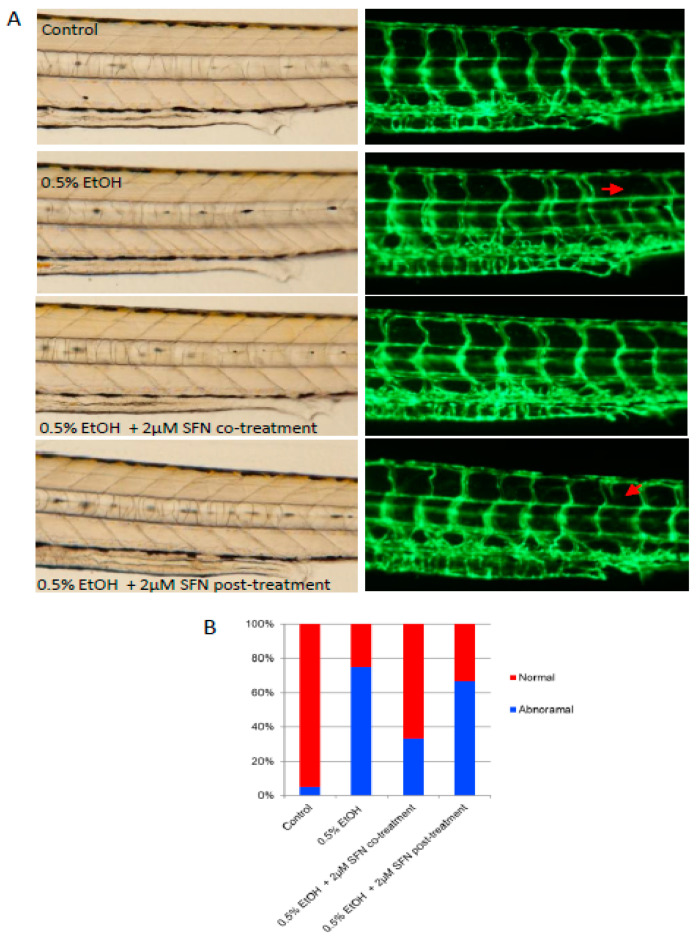
**Co-treatment and post-treatment of SFN attenuate ethanol-induced dysangiogenesis in zebrafish embryos.** (**A**) Zebrafish embryos (*Tg: fli1-eGFP*) were exposed to 0.5% ethanol during 3–24 hpf with or without 2 μM SFN during the same development phase. A group of embryos were also post-treated with 2 μM SFN after being exposed to 0.5% ethanol during 3–24 hpf. Representative images show the vascular structure in the trunk area of the embryos. Arrows indicate the malformation of the intersegmental vessels. (**B**) The bar graph shows the percentage of embryos with or without vessel malformation.

Vascular deficits have been documented in humans diagnosed with FAS [27,28]. To determine whether ethanol exposure induces vascular abnormalities in the zebrafish model and whether SFN can rescue vessel development, we exposed zebrafish embryos to a low dose (0.5%) of ethanol and either co-treated or post-treated the embryos with 2 μM SFN. The transgenic zebrafish embryos (*fli1-eGPF*) in which blood vessels are labeled by enhanced green fluorescence under the control of *fli1* promoter [29,30] were used for this study. As shown in Figure 5, ethanol exposure resulted in abnormal angiogenesis in the embryos. Specifically, the presence of undeveloped intersegmental vessels was significantly higher in ethanol-exposed embryos (75%) compared to controls (5%), suggesting a potential inflammatory occlusion of blood vessels and restriction of blood flow caused by ethanol exposure. This vascular defect was dramatically rescued by co-treatment with SFN during ethanol exposure, with over 65% of treated embryos displaying normal vessel development. Post-treatment with SFN also partially rescued the vascular abnormalities. These results indicate that ethanol-induced vascular abnormality can be effectively rescued by SFN treatment.

## 3. Discussion

There is growing evidence in the literature indicating that ethanol exposure can result in to teratogenic effects on embryos [31]. Due to their similar morphological trails to human FASD, various animal models have been well-established as models for FASD. In zebrafish embryos, it is widely documented that reduced body length, reduced brain, small eyes, small jaws, small otic vesicles, and pericardial edema are the most recognizable malformations induced by ethanol exposure [15,22]. However, there are quite a few studies about the prevention of birth defects in exposed embryos. To date, FASD is still a serious health issue in both developed and developing countries. For instance, ithe incidence of FASD in the United States is estimated to range from 24 to 48 cases per 1000 [32]. Therefore, effective therapy for FASD is highly desired. Using a zebrafish model, our findings indicate that SFN has great potential for the prevention and treatment of FASD, offering a promising new approach to addressing this complex condition.

A series of ethanol concentrations ranging from 0.5% to 1.5% were tested in this study. Ethanol concentrations higher than 1% induced dramatic teratogenic effects. Nearly all embryos exposed to 1.5% ethanol exhibited typical malformations associated with FASD. Ethanol exposure concentrations below 1% induced moderate teratogenicity, with fewer than 50% of exposed embryos displaying clear FASD phenotypes based on their appearance. Ethanol concentrations below 0.5% did not cause significant gross morphological deformities. However, the incidence of vessel abnormalities is significant even among embryos that appear to have normal body shapes. These vascular abnormalities can manifest as a range of issues, from altered blood flow to structural defects in blood vessels, contributing to the overall pathology of FASD caused by ethanol exposure.

It is widely recognized that oxidative stress plays a crucial role in FASD. [17,33,34]. Consequently, antioxidant supplementation has been considered an important therapeutic strategy for FASD prevention. Previous studies have reported that antioxidant treatments can alleviate growth retardation and other malformations induced by ethanol exposure during fetal development. For instance, vitamin C co-treatment protected ethanol-treated Xenopus laevis embryos against microencephaly and growth retardation [13]. A synthetic SOD/catalase mimetic, EUK-134, administered to ethanol-treated pregnant mice reduced the incidence of forelimb malformations in their offspring [12]. Vitamin E [14] and black ginseng [16] also inhibited ethanol-induced teratogenesis in murine embryos. Using zebrafish embryos as a model, vitamin E and Trolox partially attenuated the incidence of pericardial edema induced by 200 mM ethanol exposure during 3–24 hpf [15]. Co-treatment of 100 mM ethanol with 10 nM retinoic acid also reversed ethanol-induced developmental defects in zebrafish embryos [22]. Although direct comparisons between our study and previous reports are complicated due to differences in animal models, modes and periods of ethanol exposure, and morphological parameters evaluated, the present study demonstrated that teratogenesis induced by exposure to a high concentration (1.5%) of ethanol could be effectively mitigated by SFN treatment. SFN has been shown to protect against ethanol-induced apoptosis in NCCs by upregulating the Nrf2-mediated signaling pathway [20]. In this study, we further confirmed the protective effects of SFN on ethanol-exposed whole zebrafish embryos. The embryonic malformations induced by 1.5% ethanol exposure during the most sensitive developmental stage were alleviated by either co-treatment or post-treatment with SFN.

Dietary supplements containing SFN, found in broccoli and other cruciferous vegetables, have shown anti-cancer effects through the activation of the Nrf2 pathway [35]. This pathway induces the expression of phase II detoxification enzymes such as NAD(P)H, NQO1, heme oxygenase-1, and antioxidant proteins like SOD and catalase [36,37]. The Nrf2-mediated signaling pathway plays a crucial role in countering oxidative stress in ethanol-exposed mouse embryos and cells [17,20]. In addition to the Nrf2-mediated antioxidant response, SFN may also mitigate ethanol-induced embryotoxicity through other functional pathways. For instance, SFN can inhibit the NF-κB pathway [38], downregulate the expression of pro-apoptotic proteins such as Bid and TNF-α, and upregulate the anti-apoptotic protein Bcl-xL [39]. Collectively, these findings strongly suggest that both antioxidant and anti-apoptotic mechanisms contribute to the attenuation of ethanol-induced embryotoxicity by co-treatment and post-treatment of SFN.

In conclusion, our data indicate that ethanol-induced teratogenesis and dysangiogenesis can be attenuated by SFN supplementation during or after ethanol exposure at the gastrulation and somitogenesis stages in zebrafish embryos. This suggests that SFN has significant therapeutic potential for the prevention and treatment of FASD. Further studies should investigate the potential of SFN in preventing FASD in humans and providing early intervention for young children affected by FASD, along with more detailed investigations into the underlying molecular and cellular mechanisms. 

## 4. Materials and Methods

### 4.1. Chemicals

Sulforaphane (SFN, 1-Isothiocyanato-4-(methylsulfinyl)-butane) was purchased from LKT Laboratories, Inc. (St. Paul, MN, USA). Absolute ethanol (Molecular biology grade) was purchased from Fisher Scientific (Fair Lawn, NJ, USA). Dimethyl sulphoxide (DMSO) and other chemicals were from Sigma-Aldrich (St. Louis, MO, USA).

### 4.2. Animals

Zebrafish (wild type AB strain) were maintained in a zebrafish housing system (Aquaneering; San Diego, CA, USA) with ultraviolet- and carbon-filtered water at 28.0 ± 0.5 °C under 14:10 light/dark cycle. The dissolved oxygen value was 7.5–8.0 mg/L, pH was 7.4 ± 0.3, and conductivity was 450–550 μSiemens. Fish were fed twice a day with commercial flake food (Tetra; Daleville, VA, USA) and once a day with live brine shrimp to incite optimal egg production. Females and males, in a ratio of 1:2, were transferred into crossing tanks in the evening before spawning induction. Mating and spawning occurred when the lights were turned on the next morning.

### 4.3. Ethanol Exposure and SFN Treatment

Zebrafish embryos at the early blastula stage (2.5–3.2 hpf) were rinsed with fresh egg water three times and randomly selected and transferred to scintillation vials (Fisher Scientific; Fair Lawn, NJ, USA). Each vial contained 20 embryos and 10 mL of solution. For ethanol treatment, fish embryos were transferred into the vials containing 0.5%, 1.0% or 1.5% (*v*/*v*) ethanol during 3–24 hpf, 24–48 hpf, or 48–72 hpf. Stable ethanol levels were maintained by using the crew cap for each vial. The ethanol concentrations in the solution after the treatment were confirmed by using EnzyChrom™ Ethanol Assay Kit (Hayward, CA, USA). The measured concentration of ethanol in the fresh exposure solutions was 1.51 ± 0.01, 0.99 ± 0.04, and 0.57 ± 0.02. (*v*/*v*), and the solutions before renewal (i.e., 24 h old) contained 1.48 ± 0.02, 0.83 ± 0.08, and 0.47 ± 0.01% of ethanol corresponding to the nominal concentration 1.5%, 1%, and 0.5%, respectively.

A series of SFN concentrations ranging from 2 to 5 μM were used in the experiments. For the SFN pretreatment group, zebrafish embryos were pretreated with SFN for 21 h during 3–24 hpf, followed by ethanol exposure during 24–48 hpf, and afterward incubated in fresh egg water. For SFN co-treatment, embryos were co-treated with SFN and ethanol for 21 h during 3–24 hpf and then rinsed and incubated in egg water for 4 days. For SFN post-treatment, embryos were exposed to ethanol for 21 h during 3–24 hpf, followed by 4 days of exposure to SFN during 24–120 hpf with renewal every 24 h. Each control and treatment group consisted of four replicates (n = 20 in each replicate).

### 4.4. Observation and Measurement of Embryos

Embryos from control and treated groups were observed under a stereo microscope (Olympus IX71; Center Valley, PA, USA) and under AZ100 fluorescent microscope (Nikon, Melville, NY, USA). The body length was measured from the anterior-most point of the mouth to the posterior-most point of the tail using Photoshop 8.0 (Adobe; San Jose, CA, USA). The malformation was checked according to the phenotypes of FASD in the zebrafish model, specifically regarding facial dysmorphia (including small eyes, reduced brain, small otic vesicle, and small jaw), pericardial edema and growth retardation (reduced body length). The percentage of the embryos with moderate or severe malformations, typically associated with two or more abnormalities, in whole hatched embryos was calculated as the malformation rate.

### 4.5. Statistical Analysis

The mean differences between the treatment groups (ethanol or SFN alone) and the control group were examined using one-way analysis of variance (ANOVA), followed by Dunnett’s multiple comparison test, with a 95% confidence interval, using GraphPad Prism 10 software. An unpaired, two-tailed *t*-test was used to compare the means of the two groups. Kaplan–Meier survival analysis was determined by a Log-rank test. All values are presented as mean ± SD, and *p*-values less than 0.05 were considered statistically significant.

## Data Availability

Data is available upon reasonable request.
